# Contralateral and Ipsilateral Interactions in the Somatosensory Pathway in Healthy Humans

**DOI:** 10.3389/fnsys.2021.698758

**Published:** 2021-08-17

**Authors:** Daisuke Ishii, Kiyoshige Ishibashi, Hiroshi Yuine, Kotaro Takeda, Satoshi Yamamoto, Yuki Kaku, Arito Yozu, Yutaka Kohno

**Affiliations:** ^1^Center for Medical Sciences, Ibaraki Prefectural University of Health Sciences, Ami, Japan; ^2^Department of Cognitive Behavioral Physiology, Chiba University Graduate School of Medicine, Chiba, Japan; ^3^Department of Physical Therapy, Ibaraki Prefectural University of Health Sciences Hospital, Ami, Japan; ^4^Department of Occupational Therapy, School of Health Sciences, Ibaraki Prefectural University of Health Sciences, Ami, Japan; ^5^Faculty of Rehabilitation, School of Healthcare, Fujita Health University, Toyoake, Japan; ^6^Department of Physical Therapy, School of Health Sciences, Ibaraki Prefectural University of Health Sciences, Ami, Japan

**Keywords:** evoked potentials, somatosensory, corpus callosum, median nerve, humans

## Abstract

Hyper-adaptability, the ability to adapt to changes in the internal environment caused by neurological disorders, is necessary to recover from various disabilities, such as motor paralysis and sensory impairment. In the recovery from motor paralysis, the pre-existing neural pathway of the ipsilateral descending pathway, which is normally suppressed and preserved in the course of development, is activated to contribute to the motor control of the paretic limb. Conversely, in sensory pathways, it remains unclear whether there are compensatory pathways which are beneficial for the recovery of sensory impairment due to damaged unilateral somatosensory pathways, such as thalamic hemorrhage. Here, we investigated the interaction between the left and right somatosensory pathways in healthy humans using paired median nerve somatosensory evoked potentials (SEPs). Paired median nerve SEPs were recorded at CP3 and CP4 with a reference of Fz in the International 10–20 System. The paired median nerve stimulation with different interstimulus intervals (ISIs; 1, 2, 3, 5, 10, 20, 40, 60, and 100 ms) was performed to test the influence of the first stimulus (to the right median nerve) on the P14, P14/N20, and N20/P25 components induced by the second stimulus (left side). Results showed that the first stimulation had no effect on SEP amplitudes (P14, P14/N20, and N20/P25) evoked by the second stimulation in all ISI conditions, suggesting that there might not be a neural connectivity formed by a small number of synapses in the left–right interaction of the somatosensory pathway. Additionally, the somatosensory pathway may be less diverse in healthy participants.

## Introduction

Hyper-adaptability can be defined as the ability to adapt to changes in the internal environment caused by neurological disorders (e.g., stroke and spinal cord injury); it is essential for recovery from various disabilities. Corticospinal neurons in the forelimb region of the primary motor cortex project to contralateral and ipsilateral spinal interneurons (Yoshino-Saito et al., 2010). This ipsilateral descending pathway plays an important role in the recovery from motor paralysis after stroke (Netz et al., 1997; Levy et al., 2001). Moreover, previous studies on monkeys with spinal cord-lesions showed that the sprouting of midline-crossing axons of the corticospinal tract occurs in the spinal cord rostral to the lesion, and the sprouting was associated with improvement in hand function and locomotion (Courtine et al., 2005; Rosenzweig et al., 2010). Additionally, functional motor representation maps around the damage and remote cortical regions change with rehabilitative motor training after focal damage in the forelimb movement area of the motor cortex (Nudo et al., 1996; Frost et al., 2003; Ramanathan et al., 2006; Barbay et al., 2013). These studies suggest that a pathway normally suppressed and preserved in the course of development may participate in the control of the paretic limb to adapt to changes in the internal environment (Murata et al., 2015; Isa, 2017, 2019; Isa et al., 2019; Yamamoto et al., 2019; Kato et al., 2020).

A functional magnetic resonance imaging (fMRI) study of sensory pathways revealed that not only the contralateral primary somatosensory cortex (SI) but also the ipsilateral SI was activated following the median nerve stimulation (Nihashi et al., 2005). In macaque monkeys, bilateral receptive fields have been found in the somatosensory area 2, which is considered to be the homolog of Brodmann’s area 2 in human SI (Iwamura et al., 2001, 2002). Neural responses in the bilateral receptive fields were not found after making postcentral gyrus lesions in the contralateral hemisphere, suggesting that the neurons in the bilateral receptive fields receive the sensory information from the contralateral brain through interhemispheric transfer (Iwamura et al., 1994). The presence of a left–right interaction at various levels is important for recovery from hypoesthesia because the left–right interaction through the corpus callosum does not contribute to the recovery of hypoesthesia in cases where somatosensory pathways are damaged at the subcortical level, such as the thalamus. Several studies investigated the interactions between contralateral and ipsilateral activations using a paired median nerve somatosensory evoked potential (p-SEP) protocol (Ragert et al., 2011; Brodie et al., 2014). In this protocol, peripheral stimulation of the unilateral median nerve as conditioning stimulus (CS) always preceded the stimulation of the other median nerve as the test stimulus (TS) with some interstimulus intervals (ISIs). In the presence of a left–right interaction, the SEP evoked by the TS is attenuated by the interference of the CS. The levels of the left–right interaction in the somatosensory pathway were estimated by the length of the ISI (Ragert et al., 2011; Brodie et al., 2014). It is unclear whether the amplitude of SEPs evoked by the second median nerve stimulation is attenuated or eliminated by the first median nerve stimulation in cases of short ISIs, especially in those less than 5 ms. On the other hand, Ragert et al. showed that the first stimulation to the left median nerve decreased the SEP amplitudes (N20) evoked by the second stimulation to the right median nerve with an ISI of 20–25 ms (Ragert et al., 2011). Additionally, Brodie et al. showed that the first stimulation to the right median nerve decreased the SEP amplitudes (P14/N20 and N20/P25) evoked by the second stimulation to the left median nerve with ISIs of 25–35 and 15–35 ms, respectively (Brodie et al., 2014). These studies indicate that interhemispheric inhibitory interactions in the SI occur between the two hemispheres through the corpus callosum in the critical time interval of 20–25 ms or 15–35 ms after median nerve stimulation (Ragert et al., 2011; Brodie et al., 2014). Moreover, the results of these two previous studies are inconsistent, and there is no consensus on the ISI interval regarding the left–right interaction of somatosensory pathways. In addition, the commissural fibers between the left and right sides of the hemispheres comprise not only the corpus callosum but also other commissural fibers such as the anterior and posterior commissural fibers. It is necessary to elucidate whether there are somatosensory pathways that provide input to the contralateral SI over a long period of time through these routes. Therefore, p-SEP recordings were obtained at nine different ISIs (1, 2, 3, 5, 10, 20, 40, 60, and 100 ms) to investigate the interaction between contralateral and ipsilateral activations at various levels in healthy humans.

## Materials and Methods

### Participants

Fourteen healthy right-handed males (mean age: 30.6; range: 25–39 years) participated in this study. The total sample size (*n* = 14) was calculated using the G*Power 3.1.9.2 software (repeated measures, within factors, F-tests, effect size f: 0.25, α error: 0.05, Power: 0.8, number of measurements: 10; Faul et al., 2007). No participants had a history of neurological or psychiatric problems. The present study was approved by the Ethics Committee of Ibaraki Prefectural University of Health Sciences (approval no. 893 and e278).

### SEP Recording

**Figure 1:**
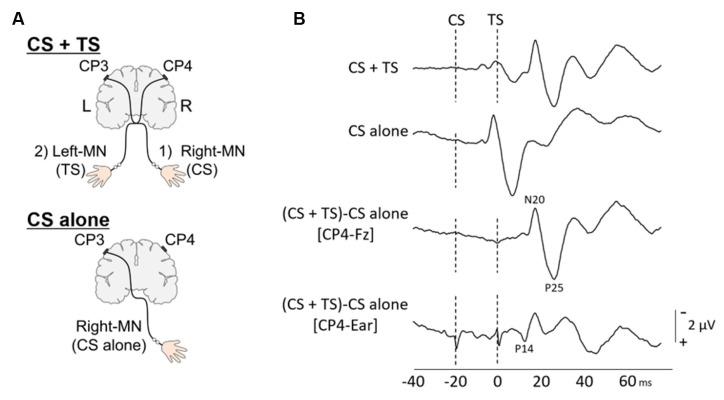
Paired median nerve somatosensory evoked potential (p-SEP). **(A)** To investigate the effect of conditioning stimulus (CS) on the SEP induced by the test stimulus (TS) (upper panel), the paired median nerve SEPs (p-SEPs) and (lower panel) the single median nerve SEPs (s-SEPs) were recorded at CP4 electrodes. In the p-SEP recording: (1) the right median nerve (right-MN) stimulation as CS always preceded; (2) the left median nerve (Left-MN) stimulation as TS with nine different interstimulus intervals (ISIs; 1, 2, 3, 5, 10, 20, 40, 60, and 100 ms). **(B)** Typical p-SEP (ISI; 20 ms) and s-SEP waveforms at CP4 from a representative individual. Dotted line: the time of stimulation. The p-SEP data were analyzed using the following formula: p-SEPs = (SEP response of CS + TS at CP4) – (SEP response of CS alone at CP4).

To investigate the interactions between contralateral and ipsilateral activations at a level below the brainstem, single median nerve SEPs (s-SEPs) and paired median nerve SEPs (p-SEPs) were recorded using a previously described protocol with minor modifications (Ragert et al., 2011; Brodie et al., 2014; Figures 1A,B). In the s-SEP paradigm, s-SEPs were obtained by stimulating the right and left median nerve at the wrist. In the p-SEP paradigm, peripheral stimulation of the right median nerve as conditioning stimulus (CS) always preceded the left median nerve stimulation as the test stimulus (TS) with nine different ISIs (1, 2, 3, 5, 10, 20, 40, 60, and 100 ms). Electroencephalogram (EEG) data from three-channel electrodes (Fz, CP3, and CP4) were recorded using a BrainAmp DC (Brain Products, Gilching, Germany) and TMS-compatible EEG electrode caps. The ground electrode was placed on the skin over the midpoint between the radius and ulna at the height of the proximal 1/2 of the right forearm, and the linked ear was used as a reference. The impedance of each electrode was set at < 5 kΩ. EEG signals were recorded at a sampling frequency of 5 kHz with a bandpass of DC–1 kHz.

A Neuropack X1 (Nihon kohden, Tokyo, Japan) was used to deliver electrical stimuli of 0.2 ms duration at a rate of 3 Hz. The stimulus intensity was set three times the perceptual threshold [7.46 ± 1.21 mA for the right median nerve, 7.58 ± 0.52 mA for the left median nerve (mean ± standard deviation SD)]. During the p-SEP condition, the right median nerve stimulation always proceeded the left median nerve stimulation using nine different ISIs (1–100 ms). During the s-SEP condition, a test stimulus (TS alone, left median nerve) and a control stimulus (CS alone, right median nerve) were applied. The order of conditions [CS + TS (1–100 ms), TS alone, and CS alone] was pseudo-randomized during the experiment. A total of five, 500 stimulus-related epochs were recorded with 500 epochs for each condition [9 ISIs (CS + TS), TS alone, and CS alone]. We asked the patients to count the electrical stimuli during SEP recordings to counteract the attention effect.

### Data Analysis

Epochs were digitally filtered using a band-pass Butterworth filter (1–200 Hz), and each condition was averaged (Ragert et al., 2011). Each SEP component was calculated by montages described below using Matlab R2019a (MathWorks, Natick, MA, United States; Mauguiere et al., 1999; Cruccu et al., 2008). The subcortical component (P14) = CP3 or CP4-linked earlobes; the cortical components (P14-peak–N20-peak and N20-peak–P25) = CP3 or CP4 − Fz. Amplitudes of the subcortical component (P14) were measured as the P14-onset to P14-peak. Amplitudes of cortical components (P14-peak–N20-peak and N20-peak–P25) were measured from peak to peak.

To evaluate the effect of CS (right median nerve stimulation) on TS (left median nerve stimulation), the p-SEP data were analyzed using the following formula:

*p-SEPs at CP3 or CP4 = (SEP response of CS + TS) – (SEP response of CS only)*

### Statistical Analyses

**Figure 2:**
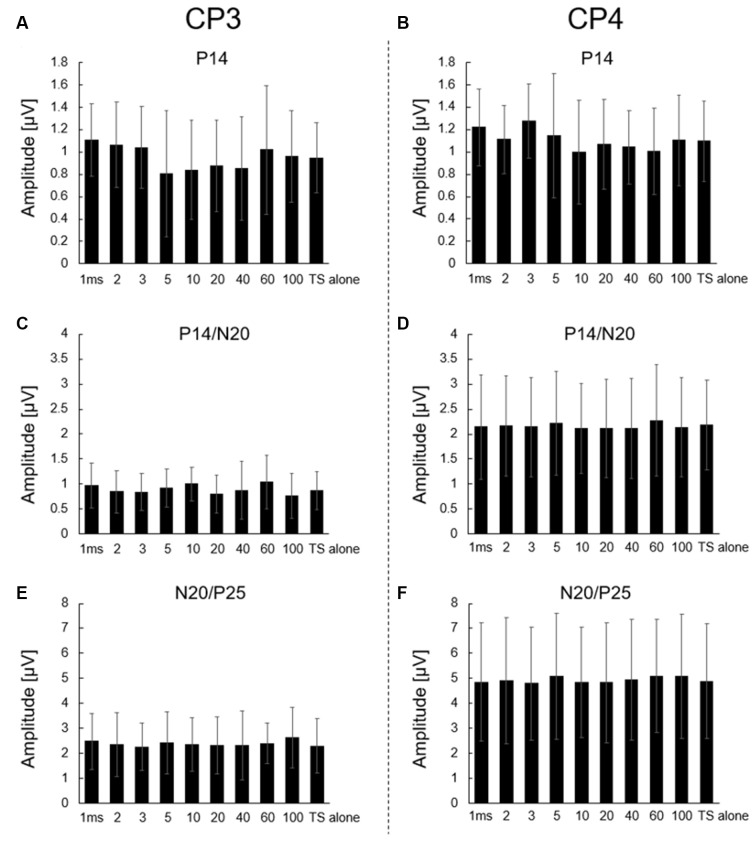
Effect of CS to the right median nerve on P14, P14/N20, and N20/P25 responses of CP3 and CP4 electrodes. Averaged **(A,B)** P14, **(C,D)** P14/N20, and **(E,F)** N20/P25 amplitudes for TS alone and all ISI conditions (1, 2, 3, 5, 10, 20, 40, 60, and 100 ms). Left panels: CP3 electrode; right panels: CP4 electrode. No significant differences were observed between each ISI condition and TS alone condition in all amplitudes. All data are presented as means ± SD.

All analyses were conducted using the SPSS 12.0 for Windows (SPSS, Chicago, Illinois). Data are shown as mean ± SD for all results (Figure 2). Data distribution was evaluated using the Shapiro–Wilk normality test. All data were normally distributed. Data of each amplitude (P14, P14/N20, N20/P25) were analyzed using one-way repeated-measures analysis of variance with factor ISI conditions (TS alone, 1, 2, 3, 5, 10, 20, 40, 60, and 100 ms). Dunnett’s test was used for *post hoc* comparisons. Statistical significance was set at *p* < 0.05.

## Results

Supplementary Figures 1–14 show the s-SEP and analyzed p-SEP (s-SEP subtracted) waveforms at CP3 and CP4 from all participants. The Shapiro–Wilk test showed that all data were normally distributed (*p* > 0.05). Figures 2A–F show no significant main effects on ISI conditions in all amplitudes at CP3 and CP4 (P14: CP3, *F*_(9,117)_ = 1.421, *p* = 0.187; CP4, *F*_(9,117)_ = 1.236, *p* = 0.280; P14/N20: CP3, *F*_(9,117)_ = 1.574, *p* = 0.131; CP4, *F*_(9,117)_ = 0.947; *p* = 0.487; and N20/P25: CP3, *F*_(9,117)_ = 0.730, *p* = 0.681; CP4, *F*_(9,117)_ = 0.938; *p* = 0.495). Dunnett’s test showed no significant differences between each ISI and TS alone condition in all amplitudes at CP3 and CP4 (*p* > 0.05).

## Discussion

In this study, we investigated the interaction between contralateral and ipsilateral somatosensory pathways in healthy humans. The major findings of this study were that the right median nerve stimulation (CS) did not interfere with SEP amplitude (P14, P14/N20, N20/P25) induced by left median nerve stimulation (TS) in all ISI conditions (1, 2, 3, 5, 10, 20, 40, 60, and 100 ms).

If a direct connection between bilateral ascending pathways exists, the amplitude of SEP induced by the second median nerve TS would be attenuated or eliminated by the refractory period under the short ISI condition (Hoshiyama and Kakigi, 2002). However, in our result, the right median nerve stimulation (CS) did not decrease the cortical and subcortical components of the SEP induced by the left median nerve TS in short ISI conditions (1, 2, 3, and 5 ms). This result suggests the absence of a neural connectivity formed by a small number of synapses in the left–right interaction of the somatosensory pathway.

Regarding our results in the middle ISI condition (10–40 ms), Ragert et al. showed decreased N20 SEP amplitude with an ISI of 20–25 ms (Ragert et al., 2011). In contrast, Brodie et al. showed decreased P14/N20 SEP amplitude with an ISI of 25–35 ms and decreased N20/P25 SEP amplitude with an ISI of 15–35 ms, respectively (Brodie et al., 2014). In the present study, the right median nerve stimulation (CS) did not interfere with SEPs induced by the left median nerve stimulation (TS) in mid-length ISI conditions (10, 20, and 40 ms). This finding is inconsistent with the results of the two previous studies, and there is no consensus on the ISI interval regarding the left–right interaction of somatosensory pathways. However, the number of trials that were averaged was different between the previous studies and the current study (Ragert et al., 150 epochs; Brodie et al., 300 epochs; current study, 500 epochs). Importantly, increasing the number of SEP recordings improves the signal-to-noise ratio, suggesting that we were able to record SEPs by median nerve stimulation with a higher signal-to-noise ratio within the measurement conditions of the current study. Whether differences in the order of stimulation to the median nerve (Ragert et al., left to right; Brodie et al., right to left; current study, right to left) may account for conflicting results remains unclear.

The commissural fibers between the left and right hemispheres comprise not only the corpus callosum but also other commissural fibers such as the anterior and posterior commissural fibers. To investigate the left–right interaction of somatosensory pathways by polysynaptic connections, we included long ISIs (60 and 100 ms) in our evaluations and found no significant inhibitory effects of the first stimulation to the right median nerve on the SEP evoked by the second stimulation to the left median nerve. This finding suggests that there may not be a pathway to input to the contralateral SI over a long period of time or that investigating the long left–right connectivity by polysynaptic connections might be difficult using the p-SEP method.

A study with cortical surface SEP recordings in patients undergoing epileptogenic tissue resection revealed that four of 41 patients showed ipsilateral SEPs, which were very localized, were of considerably lower amplitude, had a longer latency (1.2–17.8 ms), and did not show an initial negativity (Noachtar et al., 1997). The uncrossed ipsilateral afferent system from the upper extremities includes the spinoreticular and spinomesencephalic tract, which may reach the ipsilateral somatosensory cortex (Scheibel, 1984; Noachtar et al., 1997). Although any changes in SEPs could not be identified with the electrode ipsilateral to the median nerve TS, somatosensory information may actually be directly inputted to the ipsilateral cerebrum. The existence of pathways that transfer somatosensory information to the contralateral hemisphere without involving the corpus callosum is important for recovery from hypoesthesia because when somatosensory pathways are damaged at the subcortical level, such as the thalamus, left–right interaction through the corpus callosum does not contribute to the recovery of hypoesthesia. Therefore, the presence of ipsilateral ascending pathways, which are very localized and have considerably lower amplitude using a multi-channel electrode in healthy adults, should be investigated. They should also be investigated in stroke patients, in whom large-scale changes in neural networks occur.

This study has several limitations. First, we performed a total of 5, 500 stimuli to the median nerve, which may have affected the activation or inhibition of somatosensory pathways. However, to minimize the order effect, each ISI condition was randomized. Second, all participants were men. The structure and function of the brain differs between men and women (Zaidi, 2010). Therefore, future studies should investigate sex differences in the interaction between contralateral and ipsilateral activations. Finally, the CS and TS were set to the same intensity and it remains possible that CS was not strong enough to affect the ipsilateral hemisphere. Future studies will include different CS intensities.

## Data Availability Statement

The original contributions presented in the study are included in the article/Supplementary Material, further inquiries can be directed to the corresponding author.

## Ethics Statement

The studies involving human participants were reviewed and approved by the Ibaraki Prefectural University of Health Sciences Review Board (approval no. 893 and e278). The patients/participants provided their written informed consent to participate in this study.

## Author Contributions

Conceptualization: DI, KI, HY, KT, AY, and YKo. Methodology and investigation: DI, KI, HY, and YKa. Formal analysis: DI, KT, and SY. Writing the original draft: DI. Review and editing of manuscript: KI, HY, KT, SY, YKa, AY, and YKo. All authors contributed to the article and approved the submitted version.

## Conflict of Interest

The authors declare that the research was conducted in the absence of any commercial or financial relationships that could be construed as a potential conflict of interest.

## Publisher’s Note

All claims expressed in this article are solely those of the authors and do not necessarily represent those of their affiliated organizations, or those of the publisher, the editors and the reviewers. Any product that may be evaluated in this article, or claim that may be made by its manufacturer, is not guaranteed or endorsed by the publisher.
