# Supplementary material for: Contralateral and Ipsilateral Interactions in the Somatosensory Pathway in Healthy Humans
Source: Front Syst Neurosci. 2021 Aug 17;15:698758. doi: 10.3389/fnsys.2021.698758 (PMC8415971; doi:10.3389/fnsys.2021.698758)
Supplement: Supplementary file 1 [file Data_Sheet_1.PDF]

Supplementary figures

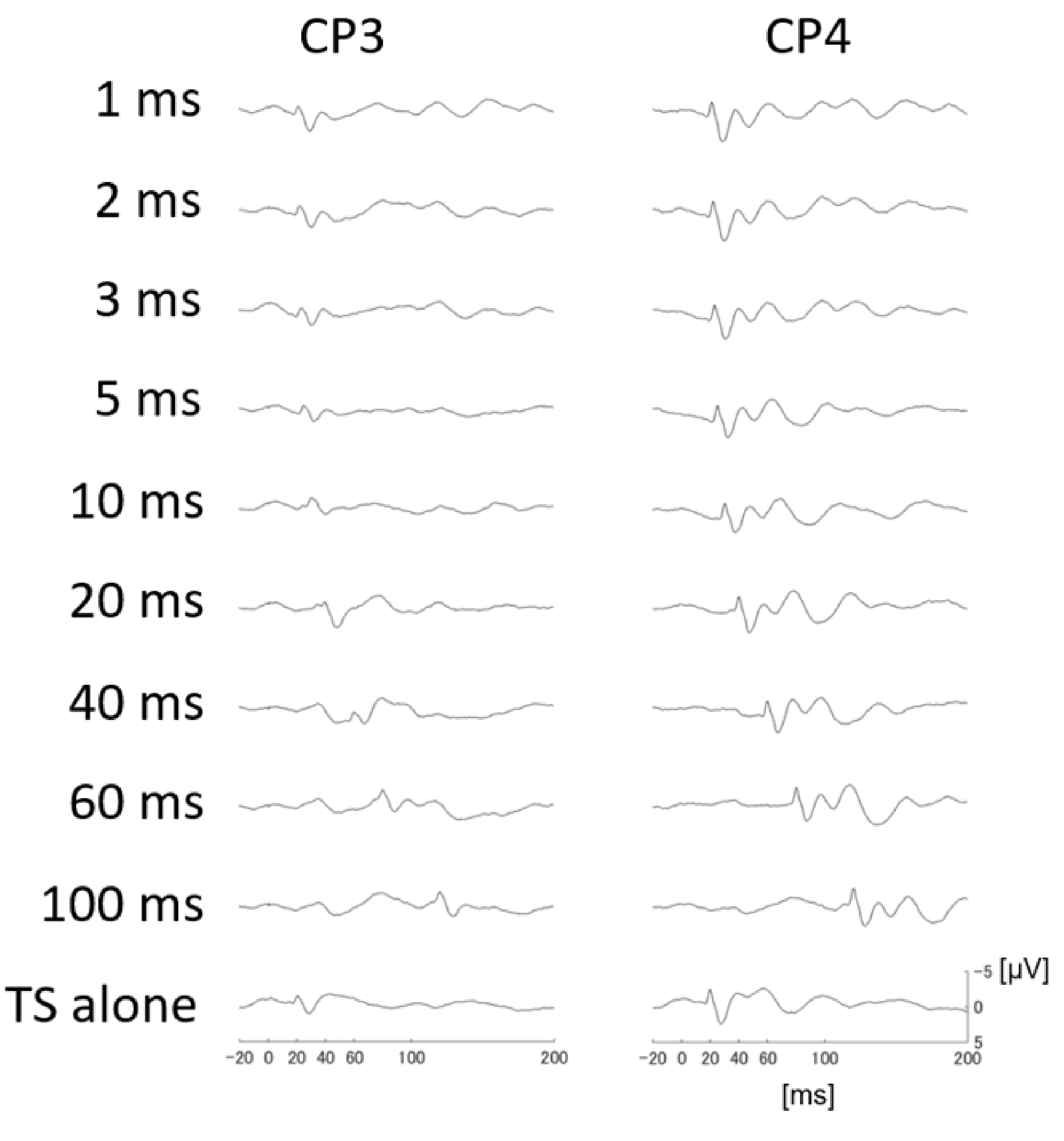

Figure S1

The s-SEP (TS alone) and the analyzed p-SEPs (s-SEP subtracted) (ISIs; 1, 2, 3, 5, 10, 20, 40, 60, 100 ms) waveforms at CP3 and CP4 from the Subject 1.

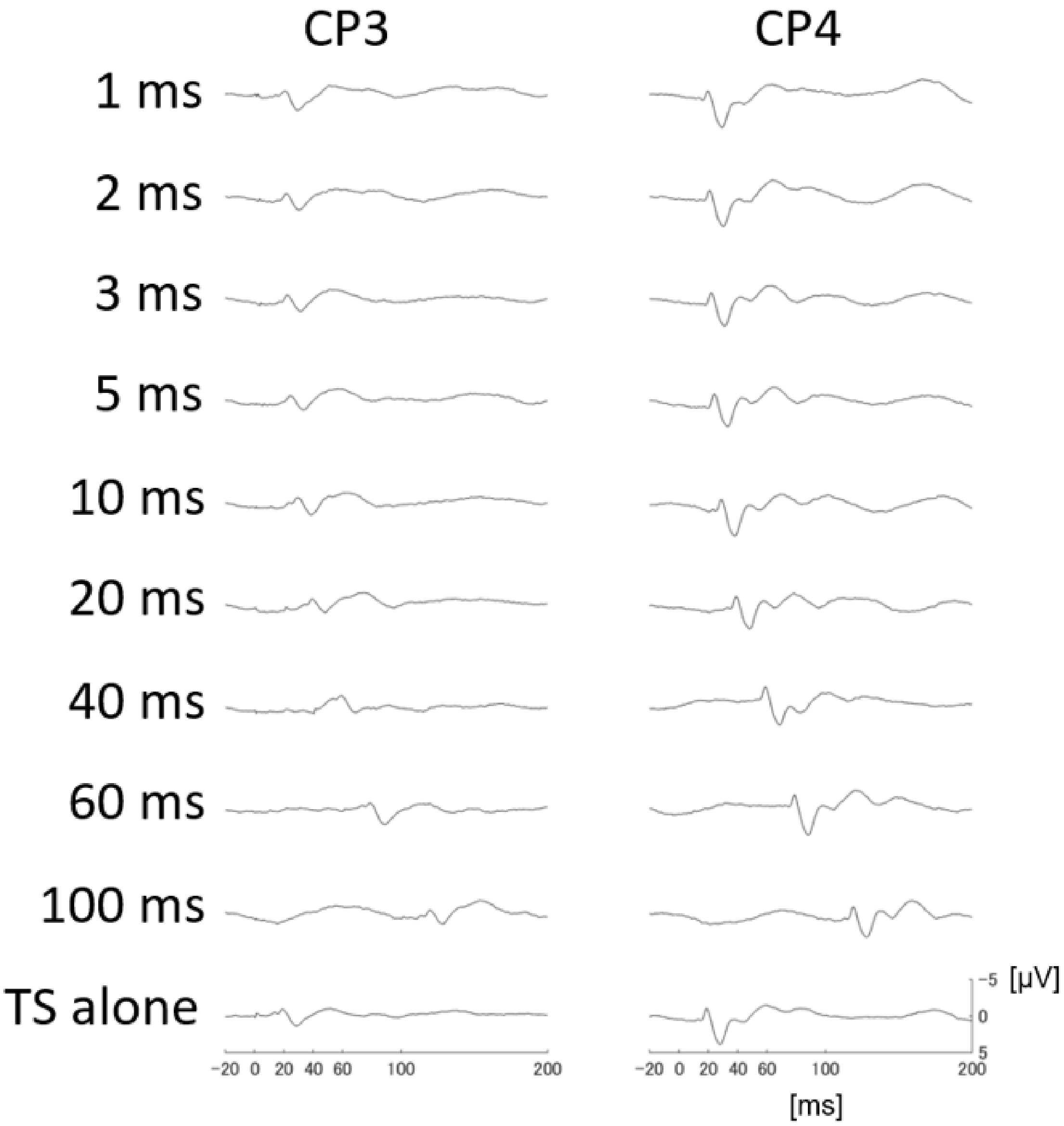

Figure S2

The s-SEP (TS alone) and the analyzed p-SEPs (s-SEP subtracted) (ISIs; 1, 2, 3, 5, 10, 20, 40, 60, 100 ms) waveforms at CP3 and CP4 from the Subject 2.

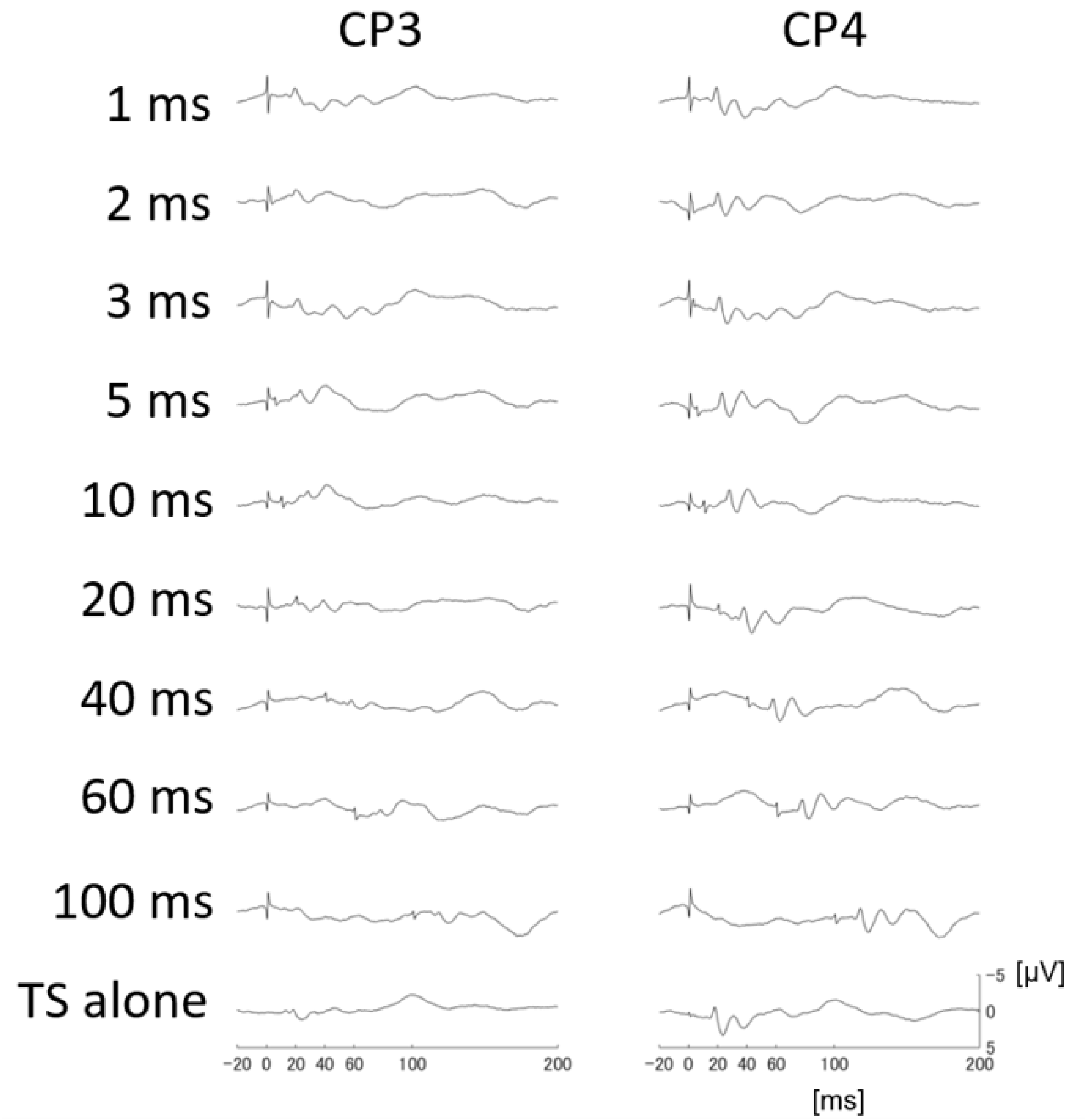

Figure S3

The s-SEP (TS alone) and the analyzed p-SEPs (s-SEP subtracted) (ISIs; 1, 2, 3, 5, 10, 20, 40, 60, 100 ms) waveforms at CP3 and CP4 from the Subject 3.

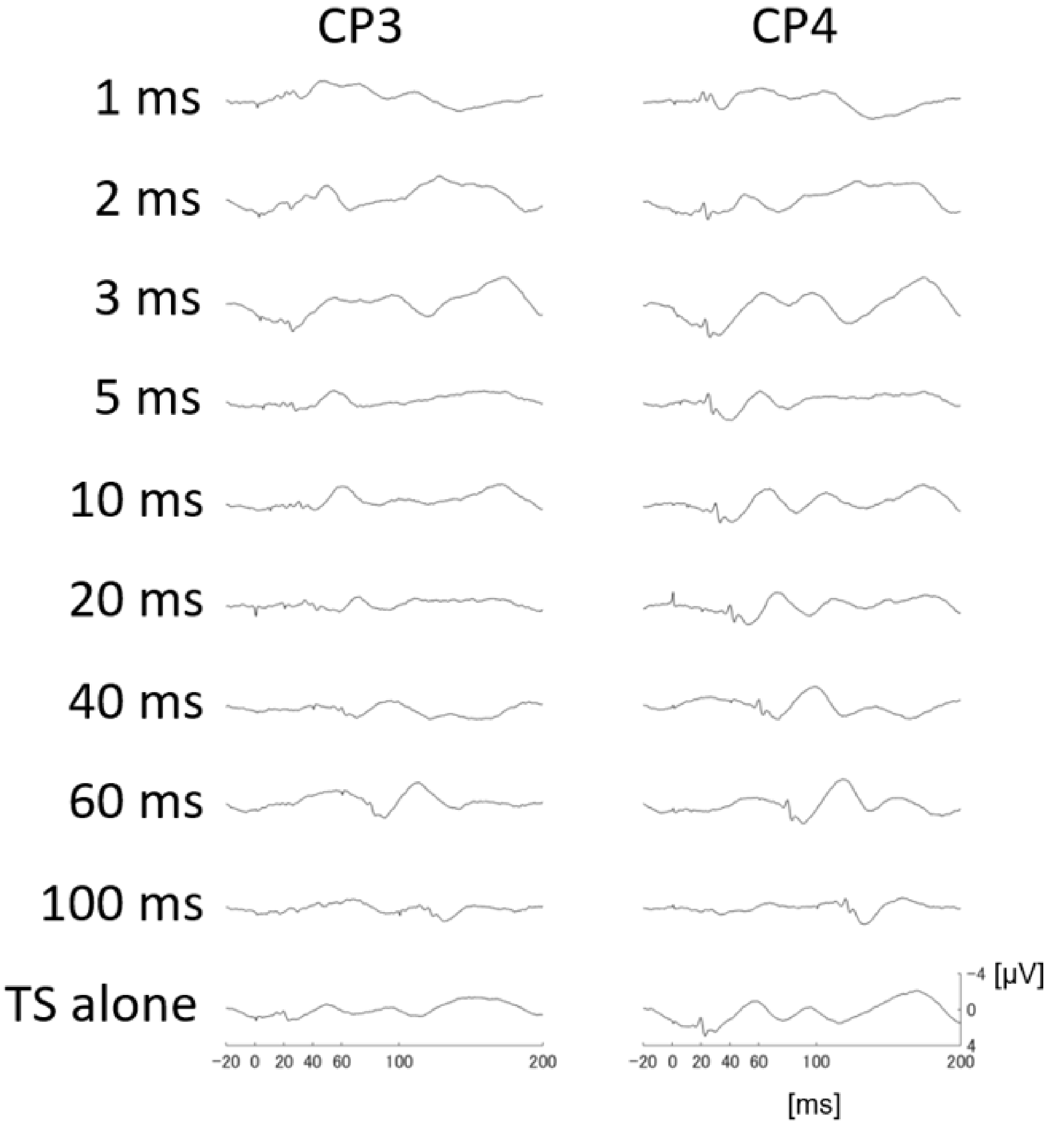

Figure S4

The s-SEP (TS alone) and the analyzed p-SEPs (s-SEP subtracted) (ISIs; 1, 2, 3, 5, 10, 20, 40, 60, 100 ms) waveforms at CP3 and CP4 from the Subject 4.

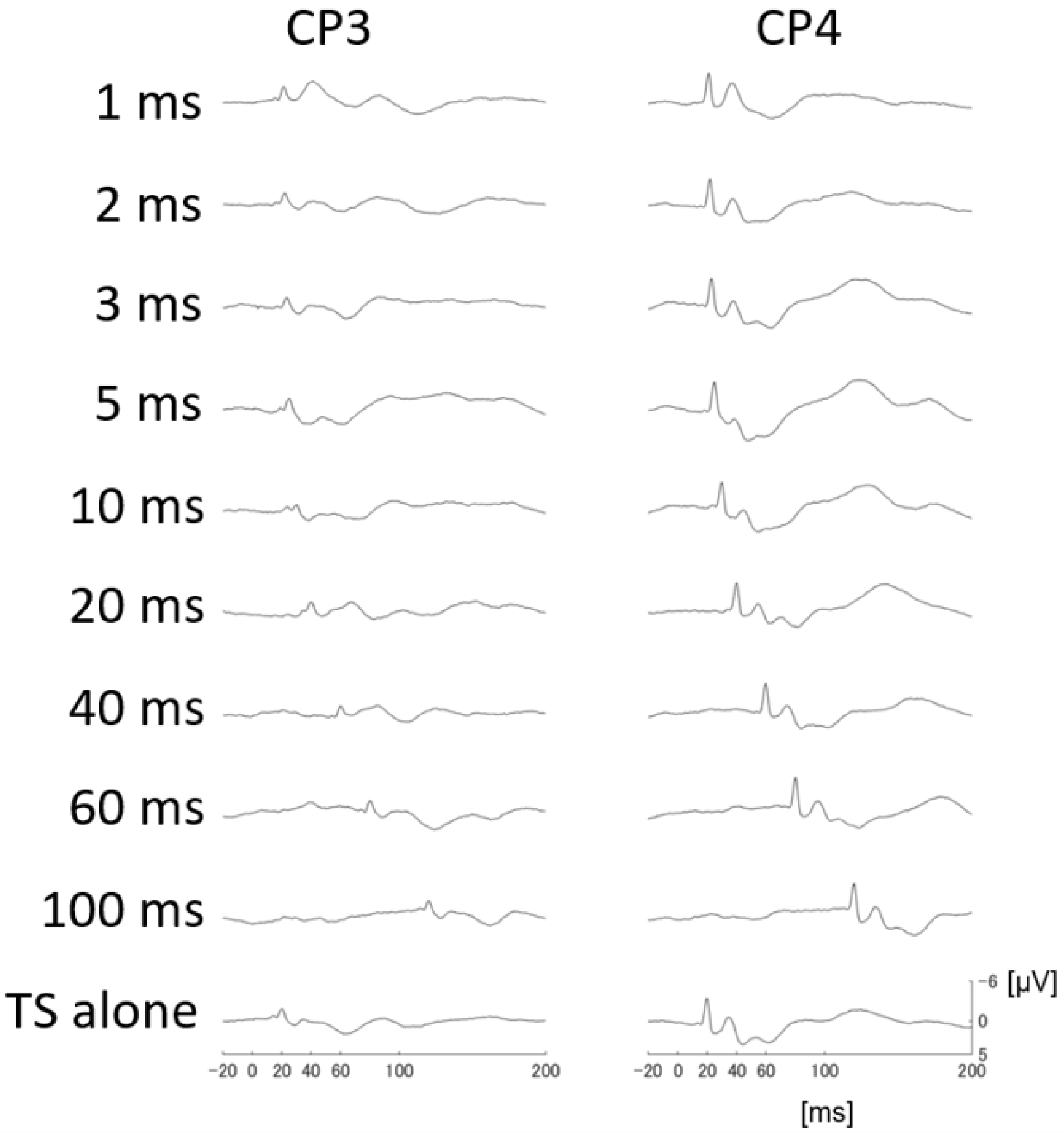

Figure S5

The s-SEP (TS alone) and the analyzed p-SEPs (s-SEP subtracted) (ISIs; 1, 2, 3, 5, 10, 20, 40, 60, 100 ms) waveforms at CP3 and CP4 from the Subject 5.

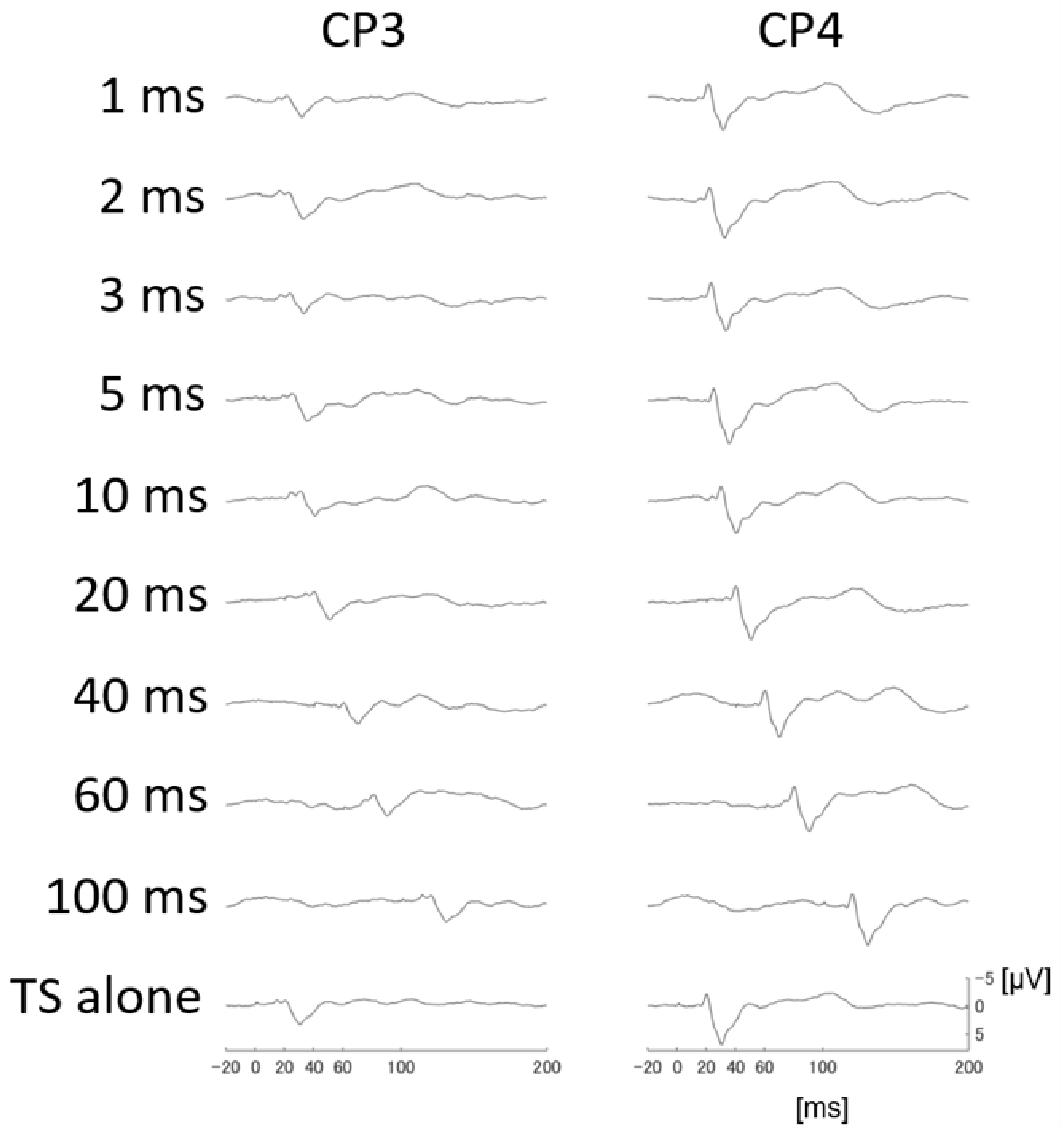

Figure S6

The s-SEP (TS alone) and the analyzed p-SEPs (s-SEP subtracted) (ISIs; 1, 2, 3, 5, 10, 20, 40, 60, 100 ms) waveforms at CP3 and CP4 from the Subject 6.

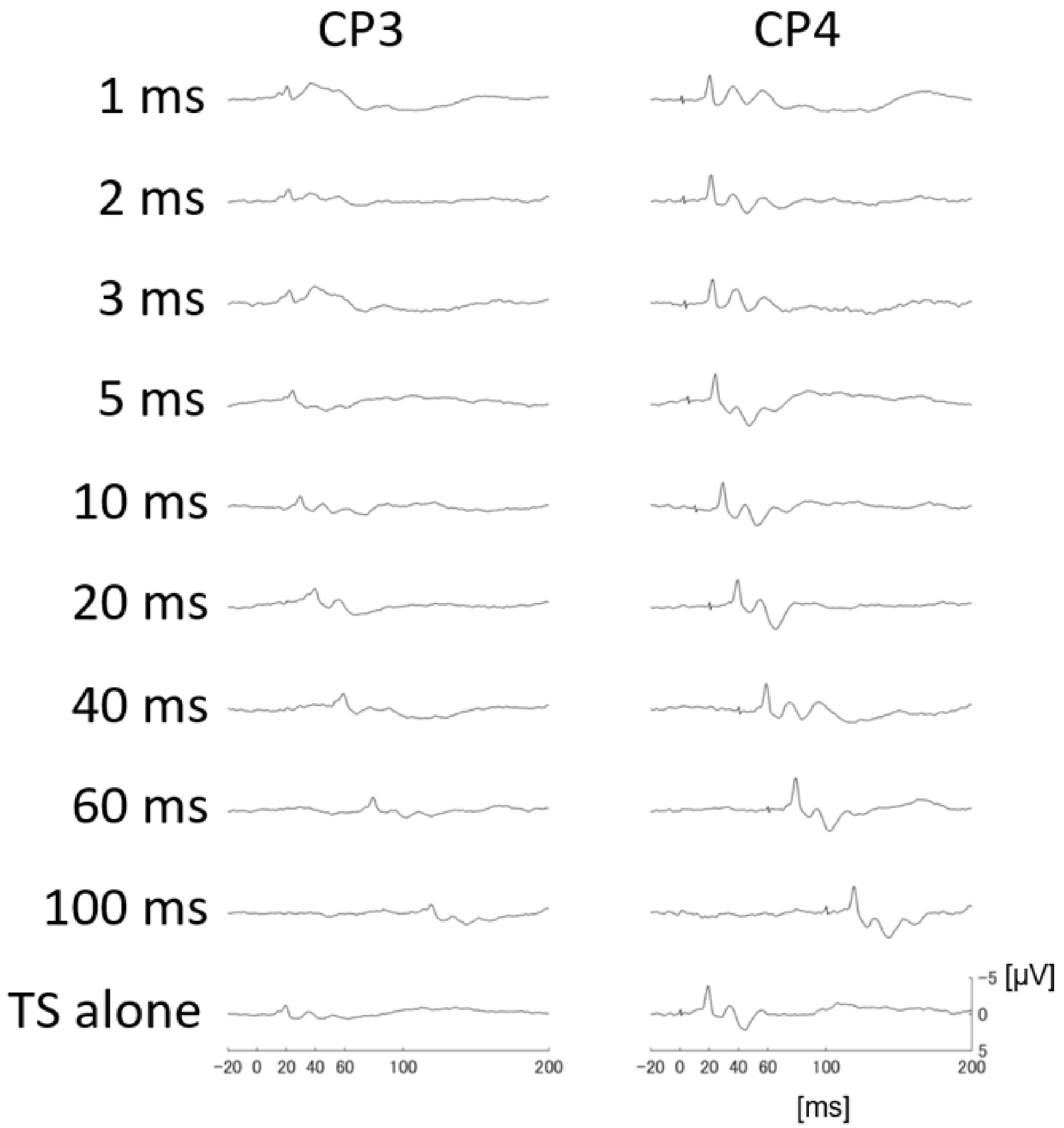

Figure S7

The s-SEP (TS alone) and the analyzed p-SEPs (s-SEP subtracted) (ISIs; 1, 2, 3, 5, 10, 20, 40, 60, 100 ms) waveforms at CP3 and CP4 from the Subject 7.

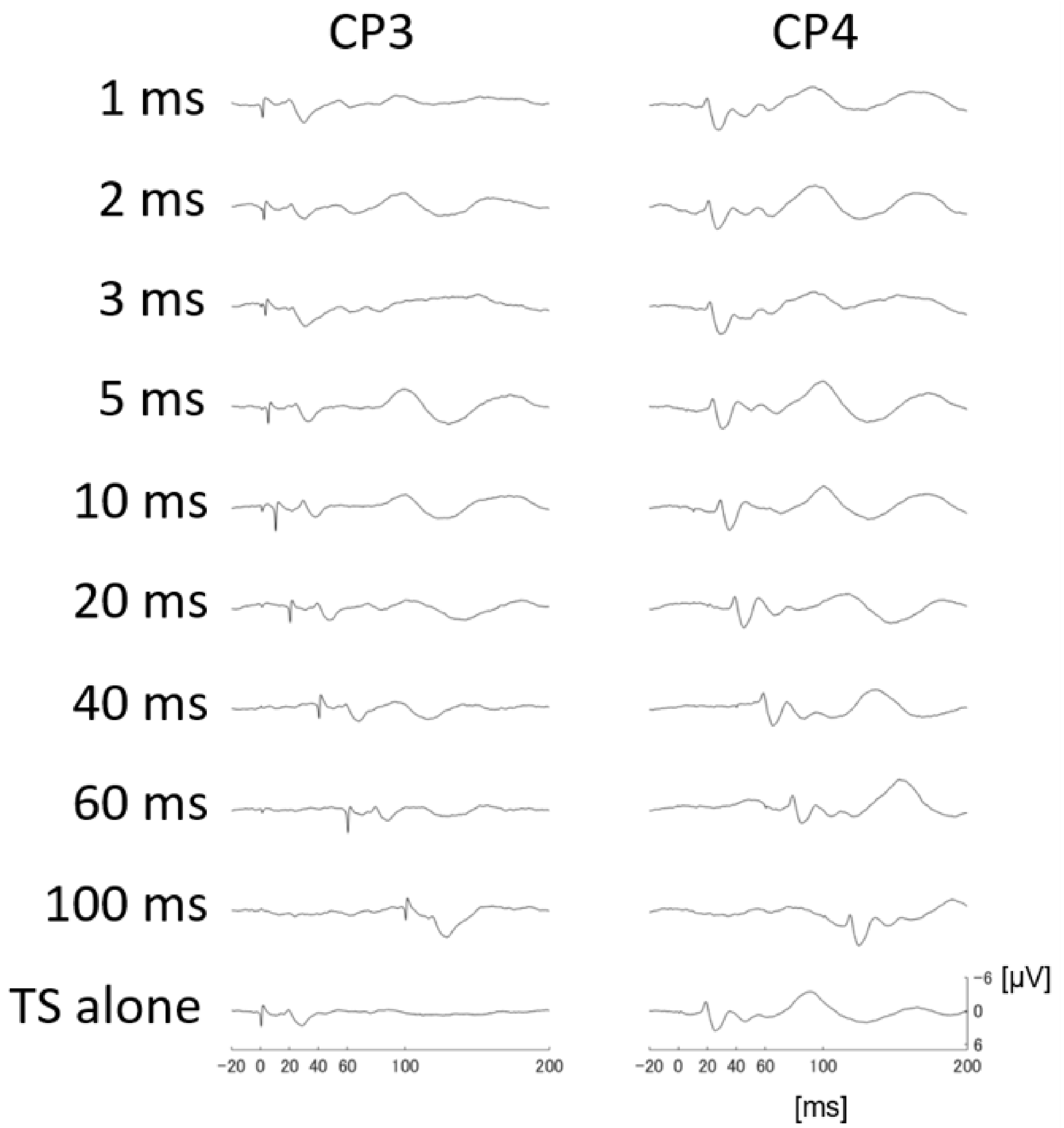

Figure S8

The s-SEP (TS alone) and the analyzed p-SEPs (s-SEP subtracted) (ISIs; 1, 2, 3, 5, 10, 20, 40, 60, 100 ms) waveforms at CP3 and CP4 from the Subject 8.

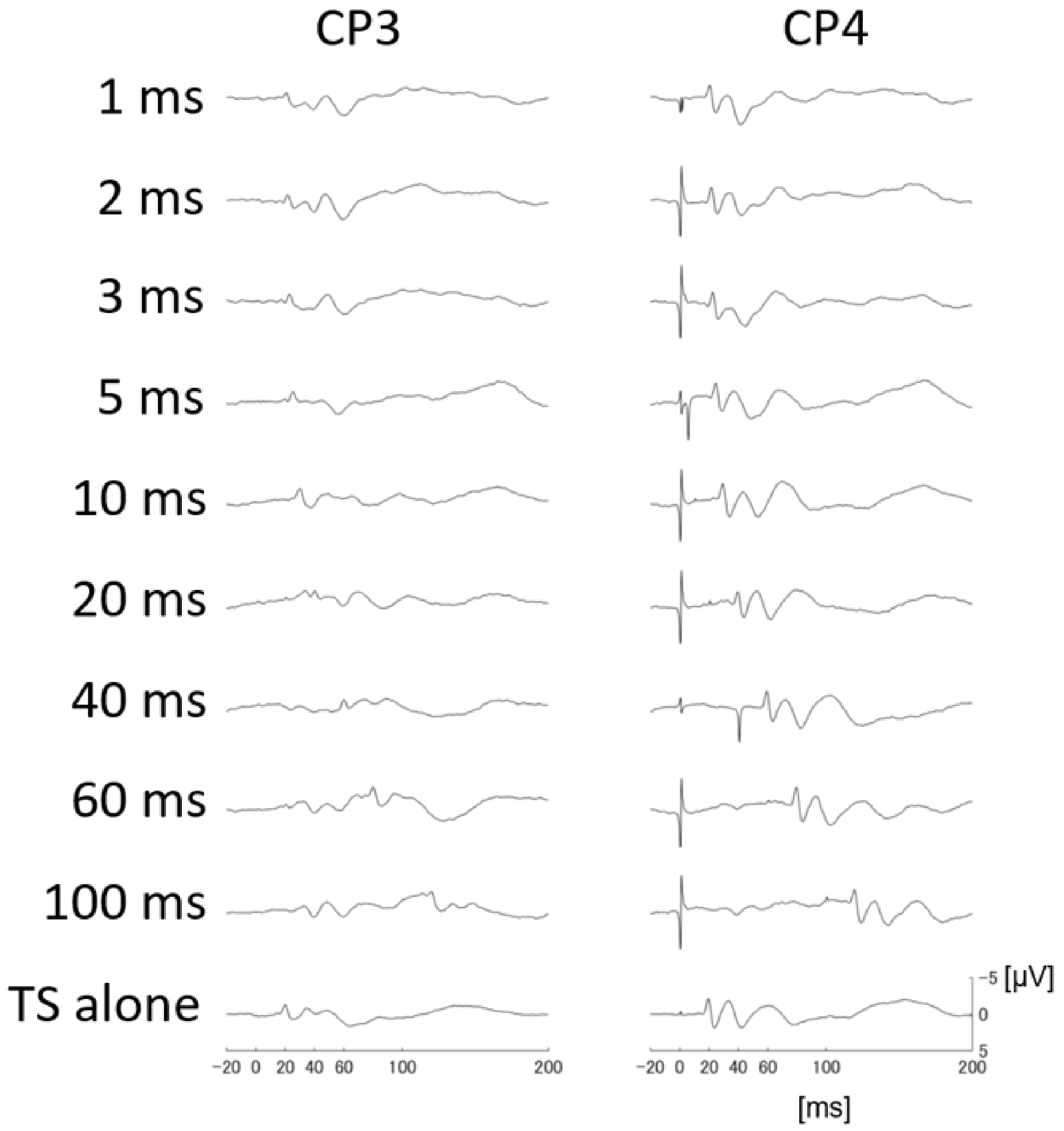

Figure S9

The s-SEP (TS alone) and the analyzed p-SEPs (s-SEP subtracted) (ISIs; 1, 2, 3, 5, 10, 20, 40, 60, 100 ms) waveforms at CP3 and CP4 from the Subject 9.

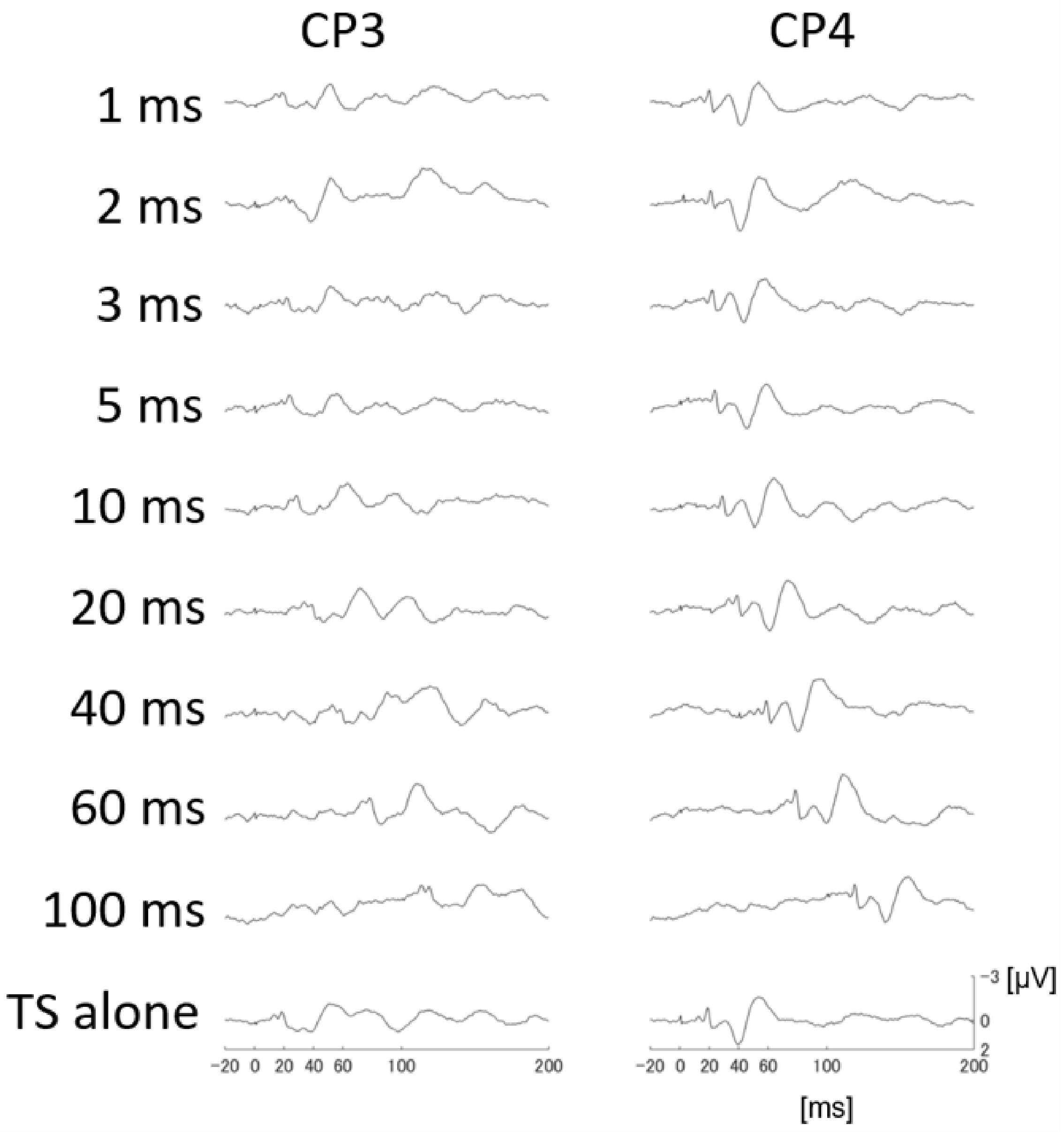

Figure S10

The s-SEP (TS alone) and the analyzed p-SEPs (s-SEP subtracted) (ISIs; 1, 2, 3, 5, 10, 20, 40, 60, 100 ms) waveforms at CP3 and CP4 from the Subject 10.

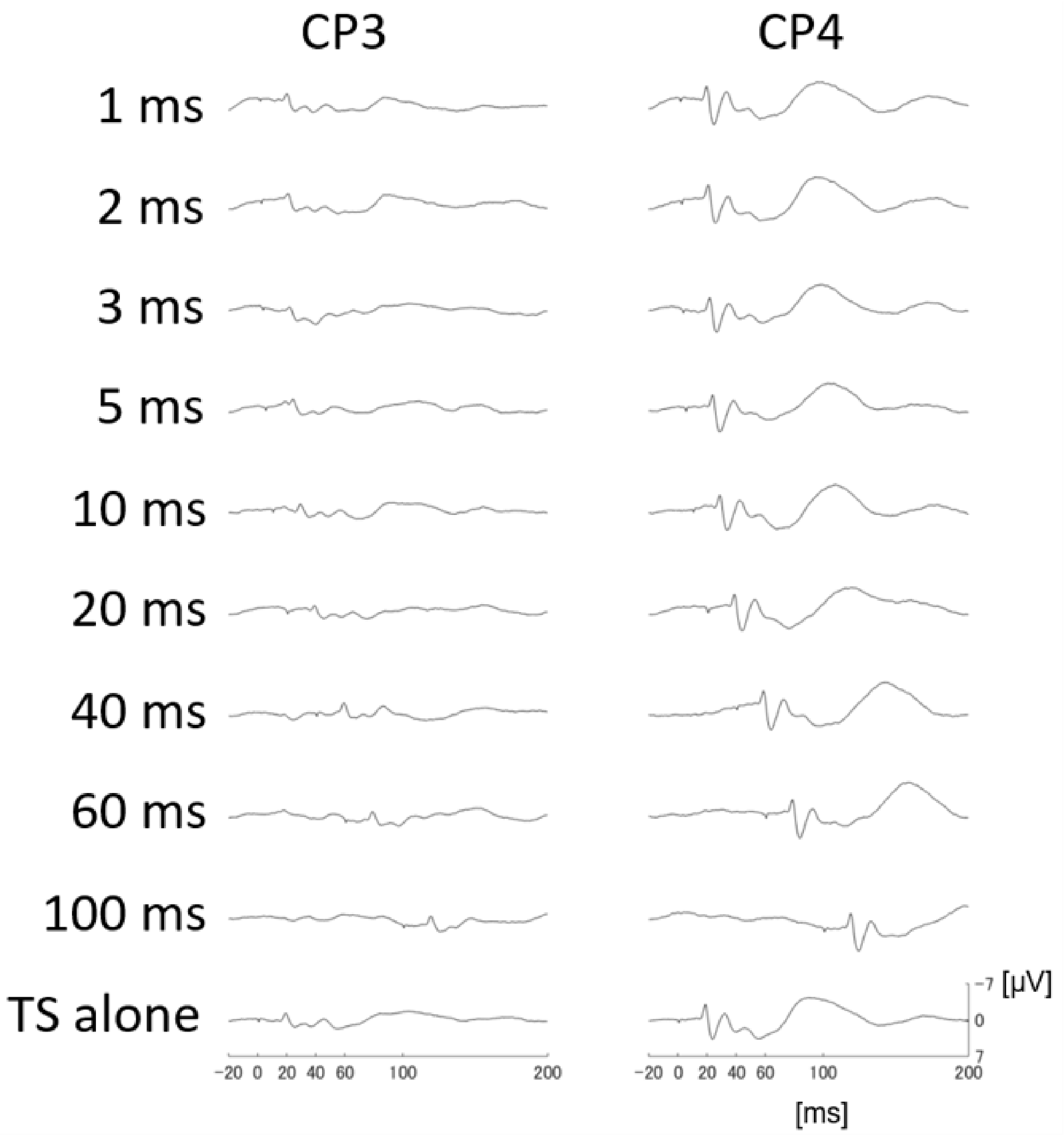

Figure S11

The s-SEP (TS alone) and the analyzed p-SEPs (s-SEP subtracted) (ISIs; 1, 2, 3, 5, 10, 20, 40, 60, 100 ms) waveforms at CP3 and CP4 from the Subject 11.

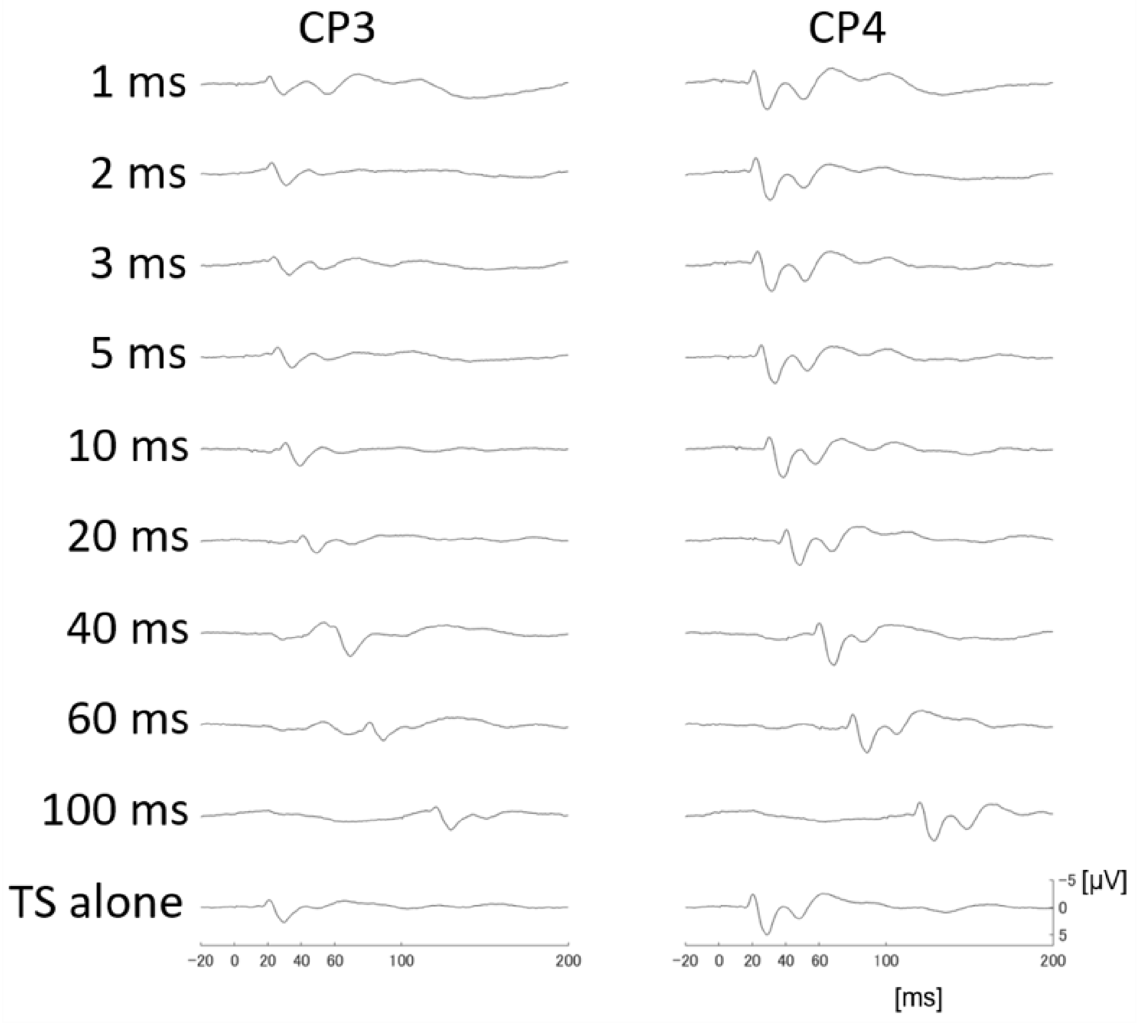

Figure S12

The s-SEP (TS alone) and the analyzed p-SEPs (s-SEP subtracted) (ISIs; 1, 2, 3, 5, 10, 20, 40, 60, 100 ms) waveforms at CP3 and CP4 from the Subject 12.

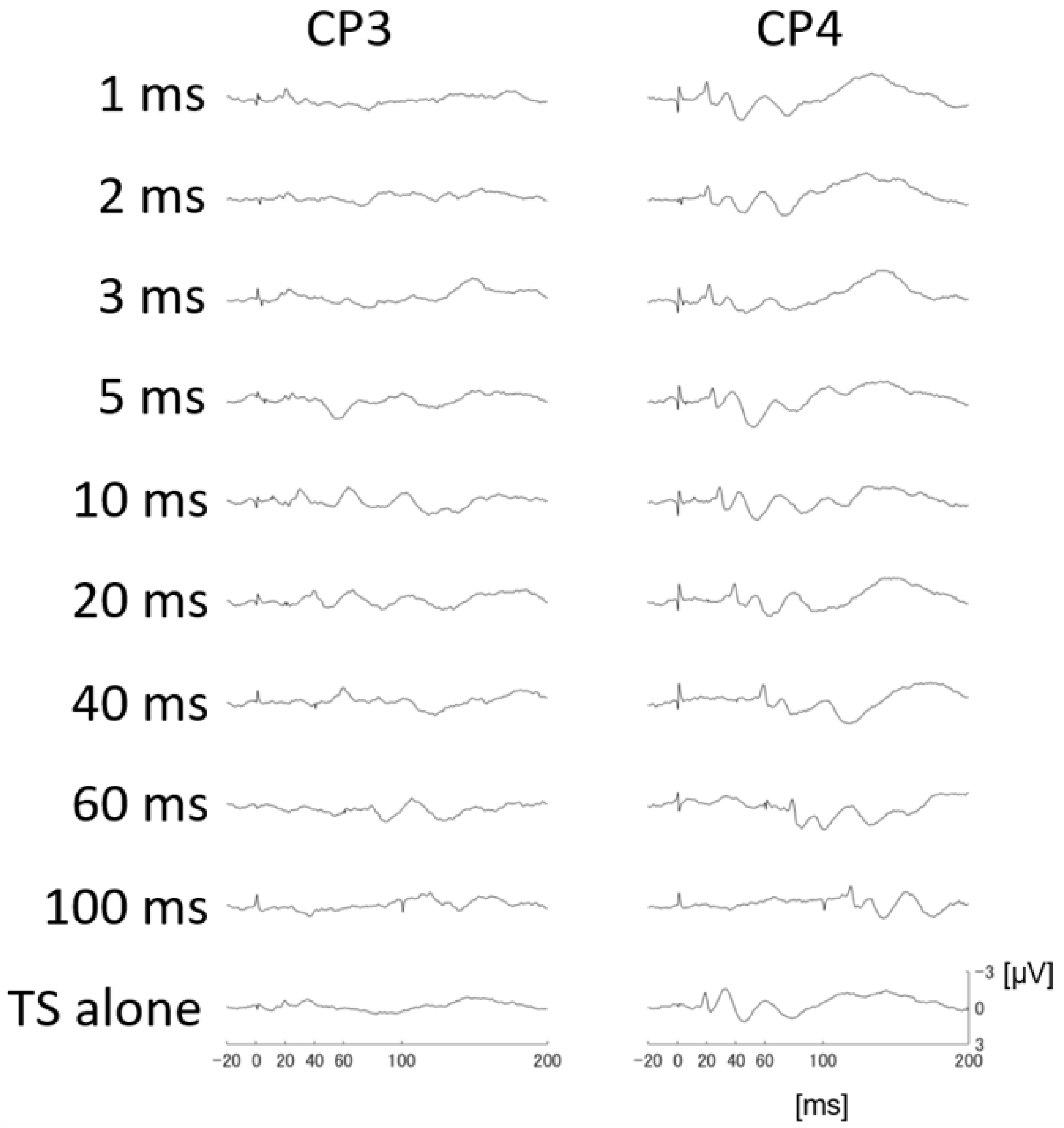

Figure S13

The s-SEP (TS alone) and the analyzed p-SEPs (s-SEP subtracted) (ISIs; 1, 2, 3, 5, 10, 20, 40, 60, 100 ms) waveforms at CP3 and CP4 from the Subject 13.

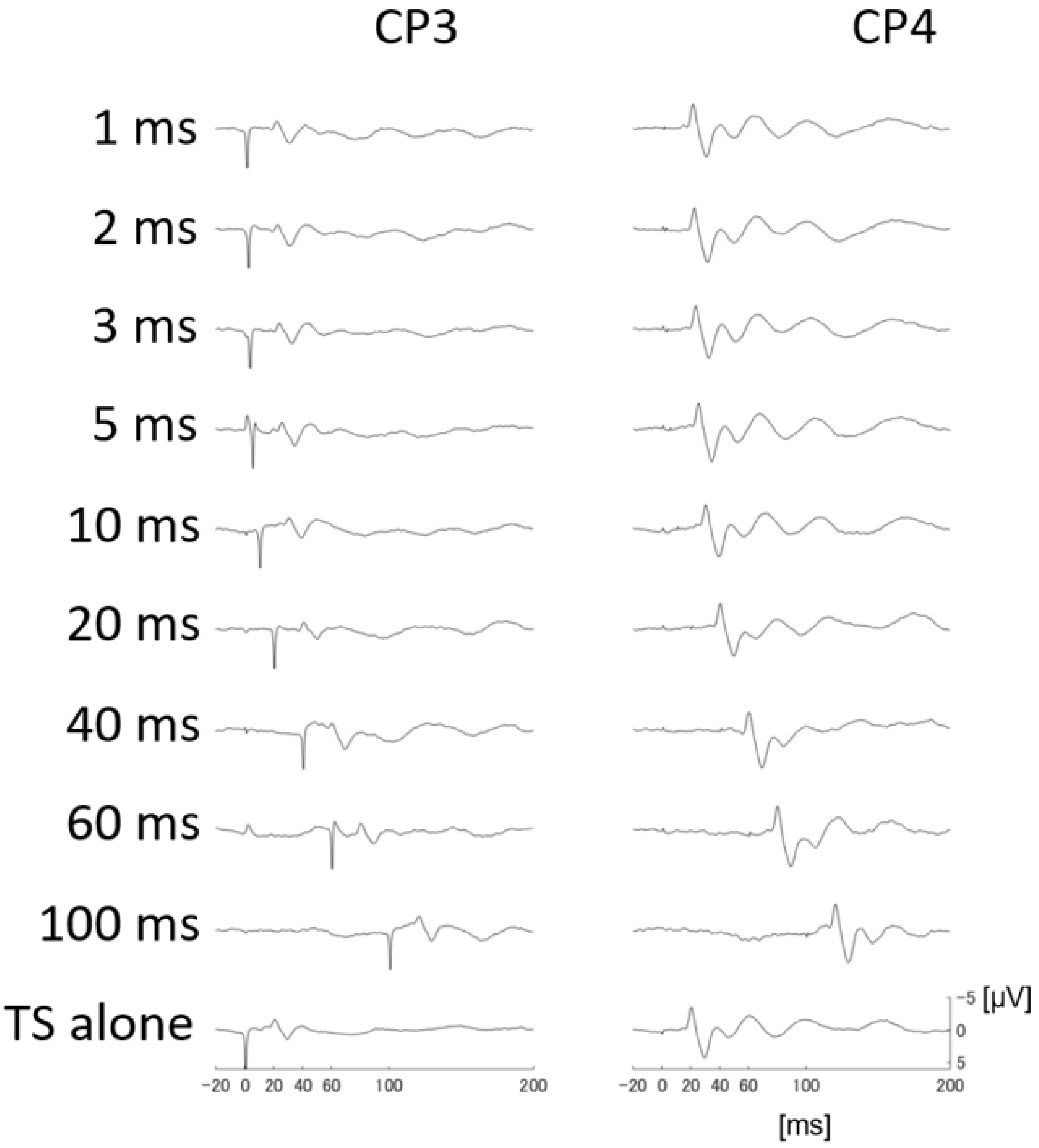

Figure S14

The s-SEP (TS alone) and the analyzed p-SEPs (s-SEP subtracted) (ISIs; 1, 2, 3, 5, 10, 20, 40, 60, 100 ms) waveforms at CP3 and CP4 from the Subject 14.
